# Standardization of pediatric uroradiological terms: a multidisciplinary European glossary

**DOI:** 10.1007/s00247-017-4006-7

**Published:** 2017-11-15

**Authors:** Pierre-Hugues Vivier, Thomas A. Augdal, Fred E. Avni, Justine Bacchetta, Rolf Beetz, Anna K. Bjerre, Johan Blickman, Pierre Cochat, Rosana Coppo, Beatrice Damasio, Kassa Darge, Alaa El-Ghoneimi, Piet Hoebeke, Göran Läckgren, Marc-David Leclair, Maria-Luisa Lobo, Gianantonio Manzoni, Stephen D. Marks, Girolamo Mattioli, Hans-Joachim Mentzel, Pierre Mouriquand, Tryggve Nevéus, Aikaterini Ntoulia, Lil-Sofie Ording-Muller, Josef Oswald, Frederica Papadopoulou, Gabriella Porcellini, Ekkehard Ring, Wolfgang Rösch, Ana F. Teixeira, Michael Riccabona

**Affiliations:** 1 Radiology, Ramsay - Générale de Santé, service de Radiologie, Hôpital Privé de l’Estuaire, 505 rue Irène Joliot Curie, 76620 Le Havre, France; 2 Pediatric Radiology, University Hospital Charles Nicolle, Rouen, France; 3 Pediatric Radiology, University Hospital of North Norway, Tromsø, Norway; 4 Pediatric Radiology, Jeanne de Flandre Hospital, Lille University hospitals, Lille, France; 5 Pediatric nephrology, Hôpital Femme Mère Enfant, Bron, France; 6 Pediatric Nephrology, Center for Paediatric and Adolescent Medicine, University Medical Clinic, Mainz, Germany; 7 Pediatric Nephrology, Oslo University Hospital, Rikshospitalet, Oslo, Norway; 8 Pediatric Radiology, Golisano Childrens Hospital, Rochester, NY USA; 9 Pediatric nephrology, Regina Margherita Hospital, Turin, Italy; 10 Pediatric Radiology, Istituto G. Gaslini, Genoa, Italy; 11 Pediatric Radiology, Children’s Hospital of Philadelphia, Perelman School of Medicine, University of Pennsylvania, Philadelphia, PA USA; 12 Pediatric Surgery and Urology, University Hospital Robert Debré, APHP, University of Paris-Diderot, Sorbonne, Paris, France; 13 Urology, Ghent University Hospital, Ghent, Belgium; 14 Pediatric Urology, University Children’s Hospital, Uppsala, Sweden; 15 Pediatric Surgery and Urology, Children University Hospital, Nantes, France; 16 Radiology, Hospital de Santa Maria, University Hospital, Lisbon, Portugal; 17 Pediatric Urology, Fondazione IRCCS Cà Granda, Ospedale Maggiore Policlinico, Milan, Italy; 18 Paediatric Nephrology, Great Ormond Street Hospital for Children NHS Foundation Trust, London, UK; 19 Dinogmi University of Genova, Pediatric Surgery and Urology, Gaslini Institute, Genoa, Italy; 20 Pediatric Radiology, Diagnostic and Interventional Radiology, University Hospital Jena, Jena, Germany; 21 Pediatric Urology, Hôpital Mère-Enfant, Hospices Civils de Lyon and Claude Bernard University, Lyon 1, France; 22 Department of Women’s and Children’s Health, Uppsala University, Uppsala, Sweden; 23 Paediatric Radiology, King’s College Hospital, London, UK; 24 Paediatric Radiology, Oslo University Hospital, Oslo, Norway; 25 Pediatric Urology, Hospital of the Sisters of Charity, Linz, Austria; 26 Radiology, Ioannina University, Ioannina, Greece; 27 Department of Pediatrics, University Hospital LKH Graz, Graz, Austria; 28 Pediatric Urology, University Medical Center Regensburg, Regensburg, Germany; 29 Pediatric Nephrology, Centro Hospitalar São João, Porto, Portugal; 30 Pediatric Radiology, University Hospital LKH Graz, Graz, Austria

**Keywords:** Imaging, Nephrology, Pediatrics, Radiology, Glossary, Urology, Standardization

## Abstract

To promote the standardization of nephro-uroradiological terms used in children, the European Society of Paediatric Radiology uroradiology taskforce wrote a detailed glossary. This work has been subsequently submitted to European experts in pediatric urology and nephrology for discussion and acceptance to improve the quality of radiological reports and communication between different clinicians involved in pediatric urology and nephrology.

## Introduction

Based on the experience of the members of the European Society of Paediatric Radiology (ESPR) uroradiology taskforce, terms commonly used in pediatric nephro-uroradiology have been (re)defined to standardize and specify terms to avoid potential misunderstandings. This work has been submitted subsequently to representatives of European Pediatric Urologists and Nephrologists for discussion and acceptance.

The purpose was not to provide in-depth explanations for all terms but effort has been made to emphasize pathophysiology illustrating the conditions when necessary. Readers should refer to textbooks or reviews for further details. We suggest no longer using some terms, which may be considered as “overused” with subsequent confusing perceptions of their actual meaning.

Other publications have previously defined some terms, particularly for duplex kidneys [1] and lower urinary tract function [2–4]. We chose to include most of these terms to provide a detailed, standardized glossary. A few terms in our glossary are not radiological terms, such as those pertaining to lower urinary tract function disorders, but have to be well known and understood by pediatric radiologists for better management of patients who are cared for by multiple clinicians. We hope this work will be helpful for a better description of pathology and for facilitation of the communication between radiologists, urologists and nephrologists.

Initially, our group faced disagreements about terms used to describe chronic urinary tract dilatation. Some members of the group added an “obstruction” value to some terms which were not considered pathological by other members. The current definition of chronic obstruction (see the detailed definition below) in practice is the one described by Koff [5] and is based on functional deterioration rather than morphologic changes. Based on this definition, we decided that none of the terms describing an upper urinary tract dilatation should have an “obstruction” connotation.

This glossary is not intended to serve as a guideline for clinical management. Other recommendations have been previously published on the management of various urological and renal disorders [6–13].

This work has been conducted in several phases. At first, some members of the ESPR uroradiology taskforce and European Society of Urogenital Radiology (ESUR) pediatric working group have searched preexisting publications about definitions of uro-radiological terms used in the pediatric field. These terms have been cited and others (re)defined. The draft has been subsequently submitted to all the members of the ESPR uroradiology taskforce for comments and editing. Thereafter, for broader comments, public presentations of this work have been performed in 2013 both at the ESPR congress (Budapest, Hungary) and at the ESUR congress (Istanbul, Turkey).

After having gathered the multiple comments and ideas in this fashion, this work has been edited and then sent to European pediatric nephrologists and urologists, who were invited based on skill set and interest to contribute to development of this glossary. After integrating their new comments and discussion of this draft among the members of the ESPR uroradiology taskforce, a new public presentations were performed at the 2015 ESPR congress (Graz, Austria) and 2015 ESUR congress (Copenhagen, Denmark) for audience input from the audience. Finally, the glossary was sent to all co-authors for final discussion and agreement before submission for publication.

## Glossary

### Acute urinary obstruction

Acute impairment to the flow of urine in the urinary tract resulting in a sudden increase in intraluminal pressure. It is often painful and is usually but not always associated with urinary tract distension.

### Adult (simple) ureterocele

The “adult” (and “simple”) part of this term should not be used any longer. It refers to a single system ureterocele which was thought to be more frequently encountered in adults and often discovered incidentally. However, ureteroceles are congenital anomalies and a ureterocele of a single system can be found in children.

### Anterior urethral valves

Anterior valves are rare and can be located anywhere distal to the membranous urethra, the peno-bulbar junction being the most common site. The exact etiology is unclear. They can be associated with a urethral diverticulum or with a Cowper’s duct cyst (syringocele). The overall urinary tract functional outcome is generally better than in the case of posterior urethral valves although it can generate urinary tract dilatation and chronic and end-stage kidney disease.

### Bifid ureter

See “incomplete ureteral duplication”.

### Bladder and bowel dysfunction (BBD)

Descriptive term of a combined bladder and bowel disturbance that does not explain pathogenesis but rather encompasses this parallel dysfunction [3]. When severe BBD results in changes in the upper urinary tract (e.g. pelvicalyceal dilatation and/or vesicoureteric reflux), it may be synonymous with the historical term “Hinman syndrome”. This latter term should be avoided. The use of the term “dysfunctional elimination syndrome (DES)” is no longer recommended.

### Bladder exstrophy-epispadias complex (BEEC)

Complex congenital abdominal midline malformation which can involve the urethra, the bladder, the pelvis, the pelvic floor, the abdominal wall, the genitalia (with frequent duplication in females), and sometimes the spine and the anus. BEEC covers a wide spectrum of anomalies of different severity levels, ranging from epispadias representing the mildest form, including proximal and distal epispadias, to the classical bladder exstrophy, and exstrophy of the cloaca, the most severe form. This latter malformation is frequently referred to as OEIS (omphalocele, cloacal exstrophy, imperforate anus and spinal dysraphism). Unlike the classical cloacal malformation which occurs almost exclusively in phenotypical girls, cloacal exstrophy is seen in both boys and girls [14, 15].

### Bladder instability

This is an old cystomanometric term which should be replaced by “detrusor overactivity” [2, 4].

### Bladder overactivity

See “overactive bladder”.

### Bolande’s tumour

See “mesoblastic nephroma”.

### Cacchi Ricci’s disease

See “medullary sponge kidney”.

### Calicectasis

See “caliectasis”.

### Caliectasis (= calicectasis)

Dilatation of one calyx or several calyces of the kidney. This term is descriptive and does not indicate associated obstruction.

### CAKUT

Congenital Anomalies of the Kidney and Urinary Tract are the commonest aetiology for children having chronic and end-stage kidney disease requiring renal replacement therapy with dialysis and/or transplantation [16, 17]. These anomalies result from aberrant ureteric bud development or from a perturbation of the reciprocal induction of the ureteric bud and the metanephric blastema during organogenesis. This interaction ensures the interactive and simultaneous development of nephrons and ureteric bud branches arising from the mesonephric duct. Renal mesenchyma condenses around the advancing bud and forms nephrons while the bud itself forms the ureter, calyces and collecting ducts. Interruption of these events results in a spectrum of disorders depending on the timing and type of interruption.

### Chronic obstruction

A restriction to urinary outflow which, left untreated, will cause progressive renal deterioration [5]. This definition applies retrospectively in practice. It is caused by intermittent episodes of acute obstruction with normal pressure within dilated urinary tract at baseline. It implies long standing impaired drainage of urine with subsequent deterioration in renal function (deterioration of glomerular filtration rate and tubular function with a decreased renal concentration capacity) and growth if left untreated. In clinical practice, this definition applies to a dilatation of at least calyces and necessitates serial functional examinations. The reference standard is renal scintigraphy. If the split renal function differs more than 5% (≤44/≥56%; ≥10% difference) in the case of bilateral single system it is considered pathologic [18, 19]. Some authors consider this threshold can be increased to 8 or 10%. An abnormal drainage pattern is no longer considered to be diagnostic of obstruction or useful for follow-up after surgery. Notice that neither the extent of the dilatation nor its morphology are taken into account in this definition. Most chronic urinary dilatations in children are not associated with a chronic obstruction as most of them will not cause deterioration of renal function. In practice the challenge remains to identify the children with an actual urinary obstruction who require surgery to prevent impairment of renal function.

### Cloaca

Normal transient embryonic cavity into which the hindgut, the genital and urinary ducts open. The cloaca is later divided into the urogenital sinus and the ano-rectal canal. Thus, a cloaca is a normal embryonic structure that should not be confused with cloacal malformation (= persistent cloaca) or cloacal exstrophy (see BEEC), which are both pathologic entities.

### Cloacal malformation (= persistent cloaca)

Abnormal persistence of the confluence of the rectum, vagina, and urethra into a single (usually obstructed) common channel. Cloacal malformation is seen almost exclusively in phenotypical females.

### Complete ureteral (= ureteric) duplication (= ureteral duplication = duplicated ureter)

Two separate ureters (with 2 separate pelvi-calyceal systems) which drain the urine of the same kidney with two ureteric orifices located in the trigone of the bladder or in an ectopic location.

### Complex ureterocele

This term should no longer be used as it implies a judgement of complexity or gravity. It has been used to describe an ureterocele in association with a duplicated kidney.

### Congenital mesoblastic nephroma

See “mesoblastic nephroma”.

### Contrast-enhanced voiding urosonography (ce-VUS)

ultrasound examination of the urinary tract with intravesical administration of ultrasound contrast agent and saline. As VCUG, it requires catheterization, but avoids radiation exposure. The diagnosis of vesicoureteric reflux is based on visualization of contrast microbubbles in the ureters or in the pelvicalyceal system. Although possible, urethral analysis remains challenging with this technique.

### Cowper’s syringocele

Tubular or cystic dilatation of a bulbourethral (Cowper) gland duct. It may communicate or not with the bulbar urethra. Syringocele can rarely generate urinary stasis or increase in pressure in the upstream urethra. Potential symptoms include obstructive voiding symptoms, haematuria, post-voiding incontinence, urinary tract infections. Syringocele may be demonstrated by VCUG, US, MRI and urethroscopy.

### Crossed renal ectopia

One (or both) kidney(s) are located contralaterally to their corresponding ureter which inserts normally into the bladder. More than 90% of crossed renal ectopia results in fusions (crossed fused renal ectopia).

### Cystic dysplasia

Presence of dysplasia (see “renal dysplasia”) in association with cysts which were absent at birth and differs from multicystic dysplastic kidney (MCDK, see this term), maybe the most severe and early form of this spectrum.

### Detrusor instability

This term should be replaced by “detrusor overactivity” (see below).

### Detrusor overactivity

Urodynamic definition corresponding to involuntary detrusor contractions during the filling phase with increased intravesical pressure. This term replaces the terms detrusor instability and bladder instability [2, 4].

### Detrusor-sphincter dyssynergia

Incoordination between detrusor and external urethral sphincter muscles during voiding (i.e. detrusor contraction synchronous with contraction of the urethral and/or periurethral striated muscles). This is seen in neurological disorders (unlike dysfunctional voiding) on urodynamic evaluation and is characterized by increased EMG sphincter activity during a detrusor contraction and by either a “spinning-top” configuration of the proximal urethra or a narrowing of the external sphincter area on voiding (or micturating) cysto-urethrography (VCUG / MCUG) or video-urodynamics [2].

### Distal

When referring to the anatomy of the urogenital tract, the natural flow of urine from the kidney to the urethra has to be considered. Proximal means close to the arriving urine unlike distal which is downstream. For example, the proximal ureter is close to the kidney whereas the distal part is close to the bladder.

### Double kidney

Inappropriate term formerly used to describe a duplex kidney (see below). The term double kidney implies two identical units while in a duplex kidney the lower moiety is embryologically larger (two thirds) than the upper (one third) [1].

### Duplex kidney (= duplex system)

Complete or partial renal duplication. It corresponds to a kidney with two pelvi-calyceal systems, with either a complete duplicated (separated) set of ureters or a bifid ureter with two proximal ureters that fuse anywhere along the course of the ureter. Ultrasonography cannot differentiate a bifid ureter from a duplicated ureter when there is no dilatation, unless it visualizes two separate bladder ostia. Imaging shows two renal sinuses separated by a parenchymal bridge. The upper part of the kidney is called upper moiety (corresponding to roughly one third of the parenchyma and one calyceal group) and the lower part or lower moiety (roughly two thirds of the parenchyma and usually two calyceal groups). The general term of duplex kidney (or system) should be avoided when possible. The terms ureteral bifidity or ureteral (or pelvic) duplication should be preferred as these two entities have usually different therapeutic consequences [1].

### Duplicated ureter

See “complete ureteral duplication”.

### Dysfunctional voiding

Intermittent and/or fluctuating flow rate due to intermittent contractions of the peri-urethral striated or levator ani muscles during voiding in neurologically normal children (unlike detrusor-sphincter dyssynergia). An assessment of urine flow with electromyography or a video-urodynamic study is required to document dysfunctional voiding. The electromyography is necessary to distinguish an interrupted or intermittent urine flow pattern secondary to a non-contractile or underactive detrusor with abdominal voiding. Dysfunctional voiding is often responsible for post-void residual urine which is a risk factor for urinary tract infection. Dysfunctional voiding corresponds to an anomaly during the voiding phase and does not apply to a disturbance of the storage phase (such as detrusor overactivity and/or incontinence) which may or may not be associated [2].

### Ectopic kidney

Kidney present in any other place than the normal location of the “renal fossa”.

### Ectopic ureteral insertion

Abnormal location of the insertion of the distal ureter. It can be intravesical (cranial or caudal to the normal opening in the trigone), but also extravesical: bladder neck, urethra, vas deferens, seminal vesicle, uterus, vagina, rectum, bowel and skin. Ectopic ureteral insertion is frequent in cases of ureteral duplication for the upper moiety ureter with usually a stenotic orifice but can also occur in a single system. In boys, ectopic ureteral insertion within the urethra is always located proximal to the external sphincter, so does not cause urine leakage. In girls, the ectopic ureteral insertion can be distal to the external sphincter and generate continuous incontinence.

### Ectopic ureterocele

Ureterocele that opens anywhere except in the normal ostium position at the lateral trigone (that would be named orthotopic). As imaging is usually inaccurate to visualize the trigone, this term can be used when the opening is anywhere else at or above the bladder neck, (i.e. bladder neck or urethra) [1]. Note that ureteroceles cannot be located cranially to the orthotopic location. Ureteroceles cannot be located elsewhere than the urinary tract.

### Enuresis

Intermittent incontinence that occurs exclusively during sleeping periods. Enuresis should not be used to refer to daytime incontinence which occurs while awake. The term “diurnal” enuresis is obsolete and should not be used any longer. Nocturnal incontinence is synonymous to enuresis [2]. Enuretic children with concomitant symptoms of lower urinary tract dysfunction differ clinically, therapeutically and pathogenically from children without such daytime symptoms. Enuresis without other lower urinary tract symptoms (nocturia excluded), and without bladder dysfunction, is defined as *monosymptomatic enuresis*. Children with enuresis and any lower urinary tract symptoms are said to have *non-monosymptomatic enuresis* [3].

### Expected bladder capacity (EBC)

Age related maximum urine volume that the bladder may contain. It corresponds to the theoretical age related expected maximum voided volume (mL) if no post-void residual urine is present. At birth, the expected bladder capacity is around 30 mL. In infants, it can be estimated by the formula 7× weight (kg). From the age of 2 years to the age of 12 years, the formula corresponds to [30 + (age in years × 30)] in mL and used as a standard for comparisons. This formula is useful from the age of 2 to 12 years, after which age EBC is at least 390 mL. Volumes are considered abnormal when they are less than 65% or greater than 150% of the expected value. The expected bladder volume is compared to the maximum voided volume (with the addition of residual urine, if present and known) [4, 20–22].

### Extra-renal pelvis

See “extra-sinusal pelvis”.

### Extra-sinusal (= extra-renal) pelvis

The major calyces open in the pelvis that protrudes outside the sinus. An extra-sinusal pelvis is frequently mildly dilated without calyceal dilatation. The dilatation occurs without any obstruction due to a high compliance.

### Floating (mobile) kidney

Positional renal ectopia corresponding to a caudal displacement of the kidney when the patient is standing up (upright).

### Horseshoe kidney

Congenital anomaly characterized by fusion of both kidneys with an isthmus of parenchymal tissue (which may or may not be functional renal parenchyma) connecting the two kidneys at the lower poles. The upper parts of the kidneys are located on each side of the spine. It is differentiated from crossed fused ectopia, in which both fused kidneys lie on one side of the spine, and the ureter of the crossed kidney crosses the midline to enter the bladder.

### Hutch diverticulum

See “para-ureteral diverticulum”.

### Hydronephrosis (= pyelocaliectasis = pelvocaliectasis = pelvicalyceal dilatation)

Dilatation of the renal pelvis and calyces. This term is descriptive and does not mean that there is an associated obstruction. However, hydronephrosis is considered to be the consequence of obstruction for many people. As a result, the use of the descriptive term pelvicalyceal dilatation should be preferred to hydronephrosis and its other synonyms. Recommended terms and terms to avoid to describe dilatation of the pelvicalyceal system and of the urinary tract are provided in Table 1.

**Table 1:** Terms related to the dilatation of the urinary tract

	Recommended terms	Terms to avoid
Dilatation of		
Calyces	Calyceal dilatation	Calicectasis Caliectasis
Calyces + pelvis	Pelvicalyceal dilatation	Hydronephrosis Pelvocaliectasis Pyelocaliectasis
Pelvis alone	Pelvic dilatation	Hydronephrosis Pelviectasis Pyelectasis
Calyces + pelvis + ureter	Ureteropelvicalyceal dilatation Ureteral and pelvicalyceal dilatations	Hydroureteronephrosis Megaureter
Ureter alone	Ureteral dilatation	Hydroureter Megaureter Ureterectasis

These terms are solely descriptive. None of them means that an obstruction is present.

An intra-uterine grading system has been developed and standardized for fetal use in 1993 by the Society of Fetal Urology (SFU). It should be emphasized that the size of the intra-renal portion of the pelvi-calyceal system is of greater importance than the extra-renal pelvis [7, 23, 24]. The existence of calyceal dilatation was also considered of greater importance than the size of the anteroposterior renal pelvis diameter (APRPD). It is noteworthy that an isolated (normal parenchyma, calyces, ureter and bladder) APRPD < 10 mm is considered normal.

The SFU classification was later re-adapted for pediatric use by the ESPR task force in 2008 [7]. A new grading system has been recently suggested by an American multidisciplinary consensus: the urinary tract dilatation (UTD) classification [25]. It focuses on pelvi-calyceal dilatation, and is based on literature review and expert opinion but is not yet validated. A prenatal (UTD A, for antenatal) and a postnatal (UTD P, for postnatal) are defined. New items include the APRPD, renal parenchyma features, ureter dilatation, bladder anomalies and oligohydramnios. Seven imaging parameters have to be evaluated for this classification:

- APRPD (on a transverse image at the maximal diameter of intrarenal pelvis)
- calyceal dilatation (central or also peripheral)
- parenchymal thickness (subjective assessment)
- parenchymal appearance: echogenicity subjectively determined by comparison with the adjacent liver or spleen, abnormal presence of cortical cysts and corticomedullary differentiation
- ureter dilatation (transient visualization of the ureter is considered normal postnatally)
- bladder anomalies (wall thickening, ureterocele, posterior urethral dilatation)
- oligohydramnios (prenatally)

The goal of this modification is to identify potential risk groups and a decision making tool. Fetuses can be in a low risk group (UTD A1) or an increased risk group (UTD A2 or A3). Children can belong to a low risk group (UTD P1), intermediate risk group (UTD P2), or a high risk group (UTD P3). The relevance of this classification has still to be validated.

### Hydroureter (= megaureter = ureterectasis)

Dilatation of the ureter (but literally refers to water in the ureter). This term should be avoided and replaced by “ureteral dilatation” (see this term).

### Hydroureteronephrosis

Hydroureter associated with hydronephrosis. This term is descriptive and does not mean that there is an associated obstruction. This term should be avoided and replaced by “ureteropelvicalyceal dilatation” or “ureteral and pelvicalyceal dilatations”.

### Incomplete ureteral (= ureteric) duplication (= ureteral bifidity = bifid ureter)

Two separate pelves and two ureters that fuse at any level proximal to the ureterovesical ostium, with a single vesico-ureteral opening.

### Incontinence

See “urinary incontinence”.

### Infundibular stenosis

Dilated calyx with or without calculi, draining through a narrowed infundibulum into a non-distended renal pelvis. It can be primary or acquired due to intrinsic narrowing such as infection (especially tuberculosis), nephrolithiasis, iatrogenic injury, and malignancy. Extrinsic stenosis can be secondary to malignancy, retroperitoneal fibrosis, or a crossing segmental artery. Similarly to the nutcracker phenomenon and syndrome, upper pole calyx dilatation related to crossing vessels may represent a normal variant without any symptom (Fraley’s phenomenon). In case of symptoms (lumbar pain, microscopic or macroscopic haematuria, nephrolithiasis, urinary tract infection) the term Fraley’s syndrome should be preferred. However, attribution of symptoms to this dilatation may be subjective.

### Intravesical ureterocele

Ureterocele that opens into the bladder higher than the bladder neck [1].

### Lower urinary tract

Bladder and urethra.

### Medullary sponge kidney (tubular pre-calyceal ectasia, Cacchi Ricci’s disease)

Usually asymptomatic, congenital condition (commonly diagnosed in young women) with diverticula and ectasia of the collecting tubules of the renal medulla. It may lead to nephrolithiasis, renal colic, urinary tract infection, hematuria, and hypertension.

### Mega(poly)calycosis

Non-obstructive dilatation of the renal calyces due to malformation of the renal papillae. The pelvis is typically normal in size. The pelvis can be slightly enlarged but with a discrepancy between the size of the pelvis and the degree of calyceal dilatation.

### Megacystis

Abnormally enlarged bladder. Bladder volume varies with age and bladder filling. References have been published in fetuses [26] and children [27].

### Megacystis-megaureter syndrome

Inappropriate term formerly used to describe high grade vesico-ureteric reflux. Megacystis-megaureter was used in cases of bilateral Grade IV or V vesico-ureteric reflux and described the morphological changes during micturition: the bladder empties normally through the urethra and abnormally through both ureters. The bladder is temporarily empty after voiding but refills very quickly by the urine that has just filled the ureters. Finally, there is chronic post-void residual urine that subsequently causes a progressive enlargement of the bladder (megacystis).

### Megaureter (= hydroureter = ureterectasis)

Ureteral dilatation (see this term). This term can be misleading as it should always be prefaced with the terms “primary” (see below), “secondary”, obstructive or refluxing (see below). Note that a combination of both mechanisms can occasionally coexist. It can be shown by a post-micturition evaluation for “trapped” urine above the vesico-ureteric junction. When describing a dilated ureter without knowing the etiology, this term should be avoided. If the cause is unknown the term ureteral dilatation (or uretero-pelvicalyceal dilatation if any) should be used (see below).

### Mesoblastic nephroma (= Bolande tumour = congenital mesoblastic nephroma)

Most common renal neoplasm in children younger than 6 months. This tumour is most of the time benign [28] and is frequently diagnosed prenatally. It cannot be differentiated from Wilms tumour by imaging alone.

### Midureteric (midureteral) stenosis

Stenosis of the midportion of the ureter. It may be caused by improper canalization, ureteral bud abnormality, congenital ureteral valves, insufficient vascular supply, congenital adhesions or postoperative strictures, retroperitoneal fibrosis, extrinsic tumoural compression, crossing vessels including a ureteral retro-caval or retro-iliac course [29].

### Multicystic dysplastic kidney (MCDK)

This is a histological definition which refers to multiple cysts with dysplasia (see “renal dysplasia”) without functioning renal parenchyma as the kidney is composed of undifferentiated and metaplastic tissues in association with cysts.

The kidney is often increased in size during fetal life and involutes during the first years of life. Rarely, the size remains stable or even increases. The ipsilateral ureter is commonly abnormal. The ureter can be atretic, absent, or with an ectopic insertion. It can be associated with an ectopic ureterocele. The ureter can also be dilated due to a possible urinary tract obstruction that can be frequently associated with MCDK. There is an increased risk of associated genital malformations.

The term MCDK should only be used when present antenatally or at birth (except for the extremely rare case of familial MCDK that develops later). Later development of dysplasia and cysts should be referred to as cystic dysplasia.

### Multilocular cystic nephroma

Benign cystic renal tumour which is often segmental and rare in children and cannot always be completely differentiated from other multilocular cystic lesions including multilocular cystic Wilms tumour and segmental multicystic dysplastic kidney.

### Nephroblastoma (= Wilms tumour)

Most common renal malignant neoplasm in childhood.

### Nephroblastomatosis

Diffuse or multifocal involvement of the kidneys by nephrogenic rests.

### Nephrocalcinosis

Microscopic (and eventually macroscopic) calcifications developing in the tubules, tubular epithelium, renal vessels or interstitial tissue. According to the anatomic area involved, it is subdivided into medullary, cortical or diffuse (global) location.

### Nephrogenic rest

Persistent embryonal metanephric blastema within the kidney. They are considered as potential precursor lesions to Wilms tumour. It is a histological definition. Macroscopic nephrogenic rests have a non-specific nodular appearance at imaging. Heterogeneous pattern or increase in size at follow-up are suggestive of Wilms tumour.

### Nephrolithiasis

Macroscopic calcification of the kidney.

### Nephroptosis (= floating kidney)

Caudal displacement of a kidney.

### Nutcracker syndrome

This term should be reserved for patients with characteristic clinical symptoms associated with demonstrable nutcracker morphologic features (also known as *left renal vein entrapment)*. This anomaly is characterized by impeded outflow from the left renal vein into the inferior vena cava due to extrinsic left renal vein compression, usually between the superior mesenteric artery and the aorta (“nutcracker”). Nutcracker morphologic features may represent a normal variant without any symptom (then called nutcracker phenomenon). It can also be associated with a retro-aortic left renal vein (“posterior nutcracker phenomenon”). Symptoms may include microscopic to macroscopic hematuria, lumbar pain aggravated by physical activity and commonly include hematuria, pain or gonadal vein syndrome (pelvic congestion syndrome), varicocele, orthostatic proteinuria, and orthostatic intolerance [30].

### Orthotopic ureterocele

Ureterocele that opens into the bladder at its normal location, i.e. on the upper and lateral aspect of the trigone. The trigone is generally not clearly seen with ultrasonography, MRI and CT. The term intravesical ureterocele should be reserved for ureterocele that opens into the bladder proximal to the bladder neck [1].

### Overactive bladder (OAB)

Urinary urgency, usually accompanied by increased frequency of micturition, with or without urinary incontinence, in the absence of urinary tract infection or other obvious pathology. The underlying condition is generally a detrusor overactivity but this term cannot be used unless invasive urodynamic investigations have been performed. OAB replaces the term bladder instability [2, 4].

### Paraureteral diverticulum (= paraostial diverticulum = Hutch diverticulum)

Congenital bladder diverticulum that occurs at the vesico-ureteric junction. They are thought to arise when bladder mucosa (urothelium) herniates through a deficient Waldeyer’s sheath and/or deficient bladder muscle at or adjacent to the ureteral hiatus. It represents a risk factor of vesico-ureteric reflux. As the herniation increases in size, the ureteral orifice subsequently becomes incorporated into the diverticulum. This extravesicalization of the intramural ureter and subsequent vesico-ureteric incompetence result in VUR.

### Pelvic congestion syndrome (PCS)

Dilated, tortuous, and congested ovarian veins produced by retrograde flow. The pathogenesis of PCS is most likely multifactorial. PCS may result from obstructing anatomic anomalies such as a retroaortic left renal vein, left ovarian vein congestion secondary to compression of the left renal vein or right common iliac vein compression. Rarely, venous compression may be due to tumours. Secondary congestion may be seen in case of valvular incompetence, portal hypertension, or acquired inferior vena cava syndrome. Symptoms of pelvic congestion are nonspecific and variable in intensity. The most common is a chronic dull and non cyclical pelvic pain.

### Pelvic kidney

Ectopic kidney found below the aortic bifurcation.

### Pelvicalyceal dilatation (= pyelocaliectasis = pelvocaliectasis = hydronephrosis)

Dilatation / Distention of the renal pelvis and calyces. The use of the simple and descriptive terms “pelvicalyceal dilatation” should be preferred to the other terms: The pelvis is wide and clearly depictable larger than 10 mm and the calyces are clearly visible. The morphology of the calyces should be described considering papillar aspects: clearly visible with a remaining concave shape towards the parenchyma, flat shape, or convex shape toward the parenchyma. Note that pelvicalyceal dilatation is not necessarily caused by obstruction.

### Pelviectasis

See “pelvicalyceal dilatation”. The use of this former term is not recommended.

### Pelvi-ureteric junction obstruction (=pyelo-ureteric/al or uretero-pelvic junction obstruction, PUJ(O)/UPJ(O))

Functional or anatomic obstruction to urine flow from the renal pelvis into the ureter at their anatomic junction. This condition is usually a congenital obstruction due to an intrinsic abnormality of collagen or muscle rather than to an extrinsic cause: crossing vessels, tumour, iatrogenic causes, inflammation, scarring and fibrosis (e.g. in high grade vesico-ureteric reflux). The use of pelvi-ureteric rather than uretero-pelvic is encouraged to respect the direction of the urine flow.

### Pelvocaliectasis

See “pelvicalyceal dilatation”. The use of this term is not recommended.

### Persistent cloaca

See “cloacal malformation”.

### Persistent urogenital sinus

Abnormal persistence of a common channel for the urinary and genital tracts due to a developmental arrest before the urogenital septum has divided the sinus into both tracts. It belongs to partial cloacal malformations and may be associated with congenital adrenal hyperplasia.

### Post-void residual urine

Residual urine is the amount of urine left in the bladder immediately after voiding. Healthy infants and toddlers have shown not to empty the bladder completely in every micturition but they do so at least once during a 4-h observation. Normal residual urine volume is zero, while 20 mL or more on repeat measurements is pathological. Values between these two measurements represent a possible clinical relevant amount of residual urine. Another easy and useful definition is the presence of a residual urine volume higher than 10% of the expected bladder volume [4, 31].

### Posterior urethral valves

Congenital obstructiing urethral membrane (rather than true valves) between the lateral urethral wall and the distal end of the veru montanum. It is caused by an abnormal migration of the Wolff channels and occurs only in males. Secondary obstruction is variable. Depending on the severity and back pressure on the kidneys during development in utero, posterior urethral valves can result in varying degrees of chronic kidney disease due to renal dysplasia.

### Proximal

When referring to the anatomy of the urogenital tract, the natural flow of urine from the kidney to the urethra has to be considered. Proximal means close to the arriving urine unlike distal which is downstream. For example, the proximal ureter is close to the kidney whereas the distal part is close to the bladder.

### Pyelectasis (= pelviectasis)

Borderline dilatation of the renal pelvis. The pelvis is measured in an orthogonal plane to the long axis of the kidney between the anterior and posterior lips (intrasinusal measurement, corresponding to an anteroposterior diameter). This measurement should not be performed outside the kidney (unless explicitly specified). The range of normal values is not straightforward and there is no simple threshold value which separates normal from abnormal. The values depend on the age, the position of the patient, the hydration status and the degree of bladder distension. Thus the use of this term may be misleading and is not recommended. For description, pelvic anteroposterior diameter should be provided in association with the grading of pelvicalyceal dilatation.

### Pyelocaliectasis

See “pelvicalyceal dilatation”. The use of this former term is not recommended.

### Pyelo-ureteral junction obstruction

See “pelvi-ureteric junction obstruction”.

### Pyohydronephrosis

Pus within a dilated renal collecting system. In practice, pyohydronephrosis can be suggested by ultrasonography when urine appears heterogeneous and relatively hyperechoic within the dilated collecting system. However, false positive cases are commonly encountered using this sonographic definition only. Severe clinical state at presentation or insufficient response to antibiotics after 48 h of antibiotics should lead to drainage when this sonographic pattern is seen. MRI has shown to be of help in showing restriction of diffusion within renal pelvis in case of pyohydronephrosis [32].

### Refluxing megaureter

Ureteral dilatation due to vesico-ureteric reflux, in other terms high grade vesico-ureteric reflux. This latter term should be preferred to avoid confusion (see “megaureter”).

### Renal agenesis

See solitary kidney. The use of this latter term is encouraged.

### Renal compensatory hypertrophy

Kidney with an increased size and a normal parenchyma appearance in comparison with nomograms associated with a pathologic contralateral kidney which is supposed to have a decreased function. The renal length centile should be greater than the height centile of the child.

### Renal dysplasia

This is a histological term with the kidney being composed, in whole or in part, of undifferentiated and metaplastic tissues. The kidney has either reduced or no glomerular function. The ipsilateral ureter is commonly abnormal. It can be atretic, absent, refluxing or with an ectopic insertion. The ureter can also be dilated due to a possible urinary tract obstruction that may or not be the aetiology of renal dysplasia. Genital abnormalities can be associated.

In radiological practice, the use of this term should be avoided. On ultrasonography, some authors have used it to describe a loss of corticomedullary differentiation with or without cysts. The kidney size may be decreased or increased during fetal life and shrinks during the first years of life. Rarely, the size remains stable or even increases. A simple description of observed abnormalities, as loss of corticomedullary differentiation, is recommended.

### Renal hypodysplasia

This is a histological term and combines a deficit in number of nephrons and undifferentiated and metaplastic tissues with reduced or no function. In radiological practice, kidneys are called “hypodysplastic” when kidneys are smaller than the normal for the age (refer to nomograms) in combination with a loss of corticomedullary differentiation. They may even exhibit cysts.

### Renal hypoplasia

This is a histological term corresponding to a kidney with a significant nephron deficit. In radiological practice, kidneys are called “hypoplastic” when they are significantly shorter than normal for the age (refer to nomograms) but retain a normal shape and corticomedullary differentiation.

### Renal moiety

Portion of the renal parenchyma in case of duplex kidney. The term “pole” should be avoided as it is often used to describe the upper and lower extremities of kidneys including those having a single system.

### Renal pole

Upper or lower extremity of renal parenchyma. This term should be avoided in case of duplex kidney for which moiety should be used.

### Retrocaval ureter (= circumcaval ureter = pre-ureteric vena cava)

Congenital disorder with a retrocaval course of the ureter due to embryological variations of the formation of the inferior vena cava. An obstruction may be associated at the crossing level.

### Segmental cystic dysplasia (= segmental multicystic dysplastic kidney)

Rare subtype of multicystic dysplastic kidney affecting a focal area of the kidney. Imaging features alone cannot completely differentiate this lesion from other multilocular cystic lesions including segmental multilocular cystic nephroma and cystic Wilms tumour, also these latter tumours are more prone to distort the kidney boundaries [33].

### Sigmoid kidney

An anomaly in which the two kidneys are fused in the form of a capital Greek letter sigma: the two renal sinuses are oriented in opposite directions. It represents a subtype of crossed renal ectopia.

### Simple (adult) ureterocele

An orthotopic ureterocele in association with a non-duplicated kidney. This term should no longer be used as it implies a judgment of complexity or gravity.

### Solitary kidney (= renal agenesis)

The radiological absent kidney may correspond to a real renal agenesis or more often results from an involution of dysplastic kidney [34]. As imaging cannot determine the cause of the missing kidney (shrinkage or renal agenesis), the use of the terms “renal agenesis” is discouraged. Müllerian anomalies are frequently associated in females. Males may also demonstrate ipsilateral mesonephric (Wolffian) anomalies.

### Spinning top urethra

Widening of the muscular segment of the urethra. This finding can be seen during the filling phase and/or the emptying phase and can be associated with dysfunctional voiding, detrusor overactivity, detrusor-sphincter dyssynergia, congenital open bladder neck anomaly or even rarely without any abnormal finding.

### Transient hyperechogenicity of the papillae

A transient increase in the echogenicity of the pyramids is commonly seen in neonates and is the result of physiologic events in the postnatal period. The maximal hyper-echogenicity is located at the apex of the pyramids or papillae, and this may extend up to approximately halfway up the pyramid. The base of the pyramid is usually spared and remains hypo-echoic. This increased echogenicity is transient and usually resolves in a few days when the infants have been rehydrated and urine output returns to standard rate. Sometimes, this hyper-echogenicity can last a few weeks. The cause was originally thought to be deposition of Tamm-Horsfall protein in the tubules or interstitium of the pyramids. However, this theory has not been confirmed and the cause of this transient increase in echogenicity in neonates remains uncertain.

### Tubular precalyceal ectasia

See “medullary sponge kidney”.

### Ucele

See “ureterocele”.

### Underactive bladder

This term is reserved for patients with low voiding frequency and a need to increase intra-abdominal pressure to initiate, maintain or complete voiding. The underlying condition is generally an underactive detrusor but this term cannot be used unless invasive urodynamic investigations have been performed [4].

### Unstable bladder

The use of this term is not recommended. The term overactive bladder (OAB) should be preferred [4].

### Upper urinary tract

Kidneys and ureters.

### Ureteral (= ureteric) bifidity (= bifid ureter = incomplete duplication)

See “incomplete ureteral duplication”. This latter term is encouraged.

### Ureteral dilatation

In practice any ureteral lumen visible without use of diuretics in the fetus, is defined as a dilated ureter. Note that recent US probes can show normal ureters up to 3–4 mm in diameter transiently (due to ureteral peristalsis) in well-hydrated neonates. The term “ureteral dilatation” is descriptive and does not mean that an obstruction is associated.

### Ureteral (= ureteric) duplication (= duplicated ureter = complete duplication)

see “complete ureteral duplication” as the latter term is encouraged.

### Ureteral fetal folds (= persistent fetal ureter = Östling embryonic folds)

Tortuosity of the proximal ureter produced by thin transverse folds due to full-thickness inward projections of the ureteral wall. They have usually no postnatal clinical significance; however, rarely they can be responsible for chronic obstruction.

### Ureterectasis (= megaureter = hydroureter)

This term should be avoided and replaced by “ureteral dilatation” (see this term).

### Ureterocele (= ucele)

Cystic dilatation of the intravesical intramucosal portion of the ureter due to a stenotic orifice of (ectopically) inserting ureter. It appears as a cystic structure within the posterior wall of the bladder. The ureter may or may not be dilated. Ureterocele is more common with ureteral complete duplication (duplex system ureterocele), typically of the upper moiety ureter with a frequent ectopic insertion although an orthotopic insertion is possible. The ureterocele is commonly orthotopic (in normal ostium position) in case of single system (single system ureterocele) [1].

### Ureterocele (= ucele) disproportion

Fluid-filled ureterocele associated with a thin ureter and nondilated upper cavities with a dysplastic or absent renal parenchyma. The term disproportion underlines that the distension of the ureterocele is usually proportional to the dilatation of the ureter and upper cavities, with the intuitive concept that a nonfunctional kidney does not produce urine and the corresponding ureterocele is collapsed. However, a tubulopathy may have been induced by a severe obstruction, and some unconcentrated primary urine coming from the involuted and dysfunctional kidney may feed the ureterocele. This entity is mainly seen in case of renal duplication, but can sometimes be observed in a single system [35].

### Uretero-pelvic junction obstruction

See “pelvi-ureteric junction obstruction”.

### Urinary incontinence

Involuntary leakage of urine; it can be continuous or intermittent. Intermittent incontinence is further subdivided into daytime incontinence and enuresis. Unlike intermittent incontinence, continuous incontinence is always pathological in any age [2].

### Urolithiasis

Macroscopic calcification in the urinary tract (see “nephrolithiasis”).

### Urogenital sinus

Normal transient embryonic sinus composed of three parts: the urinary bladder, the pelvic portion of the urogenital sinus which becomes the proximal urethra in the female and the membranous and prostatic urethra in the male, and the phallic portion of the urogenital sinus which becomes the penile urethra in the male and the vestibule and part of the urethra and vagina in the female.

### Vesico-intestinal fissure

see “cloacal exstrophy”.

### Vesico-ureteric (=vesico-ureteral) reflux (VUR)

Retrograde flow of urine from the bladder into the ureters (and potentially into the pelvo-calyceal system) due to a lack of normal valve-like mechanism of the vesico-ureteric junction. Primary VUR is common and is related to an abnormal course of the ureter within the bladder wall with a short and direct insertion, lateralization of the ostium, or immaturity of the closure mechanism. Secondary VUR is related to an increase in pressure within the bladder pertaining to bladder or urethral dysfunctions. VUR that is first identified during the filling phase is more likely to require surgical intervention. In contrast, VUR that is first identified during the voiding phase is more likely to disappear spontaneously [36]. VUR depicted by flouroscopic VCUG is classically graded according The International Reflux Study Group from grade I to V [37]:

Grade I: VUR limited to the ureter;
Grade II: VUR up the renal cavities without dilatation;
Grade III: VUR into the renal cavities inducing dilatation and eversion of the calyces;
Grade IV: VUR with moderate to marked dilatation of the ureter and pyelocalyceal system;
Grade V: VUR with marked tortuosity and dilatation of the ureter and pyelocalyceal system.

Low grade VUR (I and II) is defined by the absence of calyceal dilatation unlike high grade VUR (III-V).

A grading system that also includes possible dilatation without reflux has been developed for contrast-enhanced voiding urosonography [38].

### Voiding dysfunction

This term should no longer be used as it is a generalized name that has been popularized to denote any abnormality related to bladder filling and/or emptying. This term is not equivalent to dysfunctional voiding (or detrusor sphincter dyssynergia) [4].

### Wilms tumour

See “nephroblastoma”.
